# Clinical Outcomes Following Incomplete Arthroscopic Iliotibial Band Release for External Snapping Hip Syndrome: An Observational Study

**DOI:** 10.1111/os.14050

**Published:** 2024-03-31

**Authors:** Eic Ju Lim, Ji Wan Kim, Chang Hyun Doh, Chul‐Ho Kim

**Affiliations:** ^1^ Department of Orthopedic Surgery Chungbuk National University Hospital, Chungbuk National University College of Medicine Cheongju Republic of Korea; ^2^ Department of Orthopedic Surgery Asan Medical Center, University of Ulsan College of Medicine Seoul Republic of Korea

**Keywords:** Arthroscopy, External snapping hip, Iliotibial band release, Incomplete

## Abstract

**Objectives:**

Arthroscopic treatment is indicated for external snapping hip (ESH) syndrome in patients refractory to conservative treatment, but snapping does not disappear completely in some case. No previous studies have described the clinical course of ESH syndrome in patients who presented with persistent snapping after an arthroscopic procedure. We demonstrated the clinical outcomes following an incomplete arthroscopic iliotibial band (ITB) release for ESH syndrome.

**Methods:**

This retrospective observational study was performed at two teaching hospitals between October 2015 and December 2021. We reviewed the data of 33 patients (34 hips) aged ≥18 years, diagnosed with ESH syndrome, who were treated with arthroscopic ITB release. Patients who presented with persistent snapping despite sufficient arthroscopic ITB release following systematic order were defined as having an incomplete release. We collected the data for recurrent symptomatic snapping hip as the primary outcome after a minimum 2‐year follow‐up. The visual analogue scale (VAS) and modified Harris hip (mHHS) scores were measured as secondary outcome.

**Results:**

“Incomplete release” was identified in three of the 34 hips (8.9%). Cases of recurrent symptomatic snapping or conversion to open surgery were not observed. The symptoms of residual snapping spontaneously disappeared in all cases following routine postoperative rehabilitation within a 3‐month follow‐up period. The VAS (4 ± 1) and mHHS (17 ± 6) scores of all the patients improved.

**Conclusion:**

When refractory ESH syndrome is identified during arthroscopic surgery, appropriate ITB release and removal of the major lesion causing snapping are expected to lead to resolution of residual symptoms without conversion to open surgery.

## Graphical abstract

Natural course of incomplete release of external snapping hip. When refractory external snapping hip (ESH) syndrome is identified during arthroscopic surgery, appropriate iliotibial band (ITB) release and removal of the major lesion causing snapping are expected to lead to resolution of residual symptoms without conversion to open surgery.

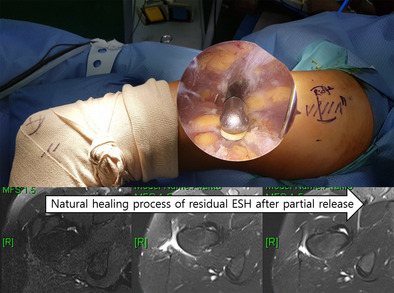

## Introduction

It is widely accepted that surgical treatment is indicated for external snapping hip (ESH) syndrome in patients refractory to conservative treatment.^1^, ^2^, ^3^ The symptoms of ESH syndrome are prominent in patients who undergo surgical treatment; thus, we can easily palpate the snapping and directly identify the resolution of snapping intraoperatively.

Presently, ESH syndrome is treated arthroscopically,^4^, ^5^, ^6^ focusing on releasing the tension in the iliotibial band (ITB) and the anterior portion of the gluteus maximus. As an alternative treatment method for open surgery for ESH syndrome, arthroscopic surgery has yielded excellent results, not only in primary cases but also in cases of revision surgery as the solution following failed open surgery techniques.^7^, ^8^ To date, several arthroscopic surgery techniques have been used,^3^, ^9^, ^10^, ^11^, ^12^ and the success rate of treatment has been reported as excellent, ranging between 87% and 100%, according to the latest review article.^13^ However, despite the obvious clinical presentation and straightforward technique, arthroscopic surgery may not effectively treat all patients with ESH syndrome. Snapping does not disappear completely, even after extended release is performed in some cases. In such situations, decision‐making regarding conversion to open surgery is difficult. Notably, conversion to open surgery is not always an easy option for surgeons. Moreover, even with the implementation of open ITB release for ESH syndrome, a recurrence rate of up to 11% has been reported.^14^

To our knowledge, no previous studies have described the clinical course of patients with ESH syndrome who remain symptomatic after arthroscopic surgery. This study demonstrated the clinical outcomes following an incomplete arthroscopic ITB release for ESH syndrome. The purpose of present study is to describe surgical technique of ITB release for ESH syndrome and evaluate the natural course of patients who presented with persistent snapping after an arthroscopic procedure.

## Methods

### Study Population

This retrospective observational study was conducted using medical records from two teaching hospitals, involving patients who underwent arthroscopic ITB release surgery between October 2015 and December 2021. The study was approved by the institutional review board (IRB No. 2024‐0050). We included adult patients (≥18 years) diagnosed with ESH syndrome who underwent arthroscopic ITB release surgery. We excluded patients who: (i) had been previously diagnosed with hip disease on the ipsilateral side; (ii) had a history of index hip surgeries; and (iii) were lost to follow‐up within 2 years. Finally, 33 patients with 34 hips were enrolled in the study.

### Preoperative Preparation

**FIGURE 1:**
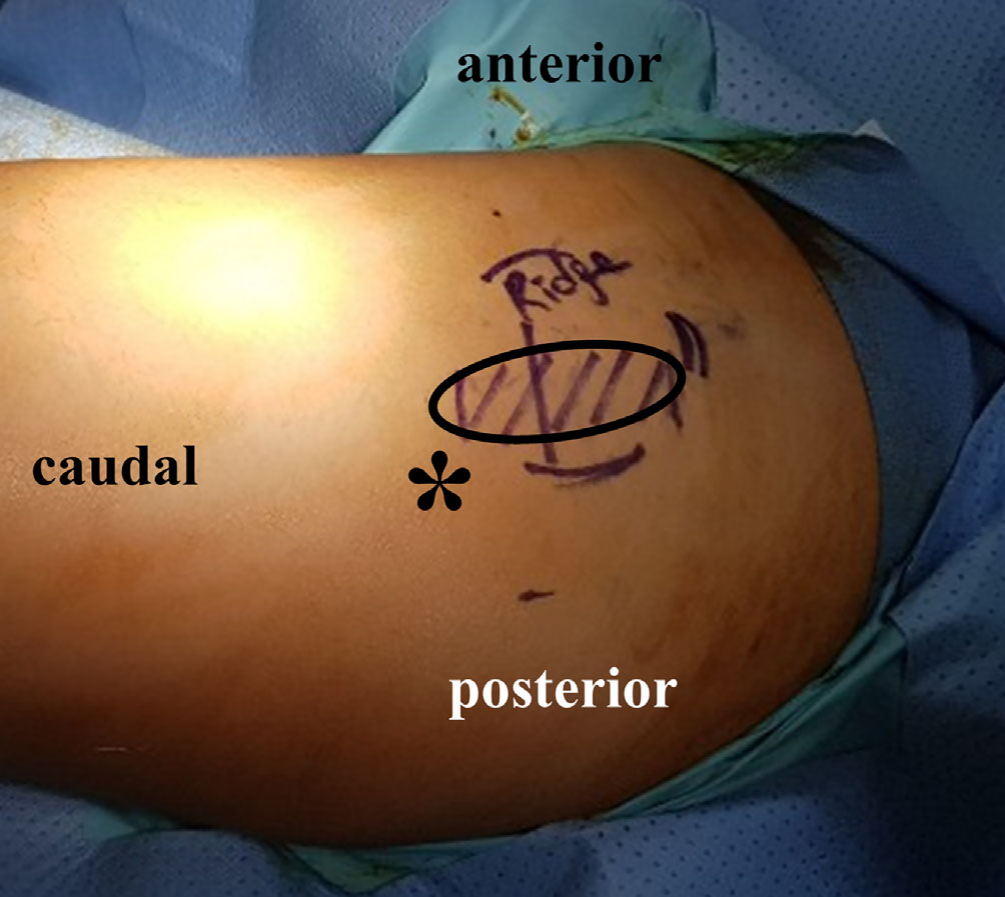
Image showing a patient placed in the lateral decubitus position. The margin of the area showing snapping is marked preoperatively (asterisk).

Snapping was re‐evaluated in all patients upon admission. In the same position of the operating table, hip flexion‐extension and adduction‐abduction exercises were performed in the lateral decubitus position to accurately confirm and demarcate the snapping site. A surgical marking pen was used to mark the boundaries of the zone showing snapping (Figure 1). Patients in whom the margins were not clearly palpable underwent ultrasonography to accurately demarcate the margin of the snapping zone over the greater trochanter (GT) of the femur.

### Surgical Procedures

**FIGURE 2:**
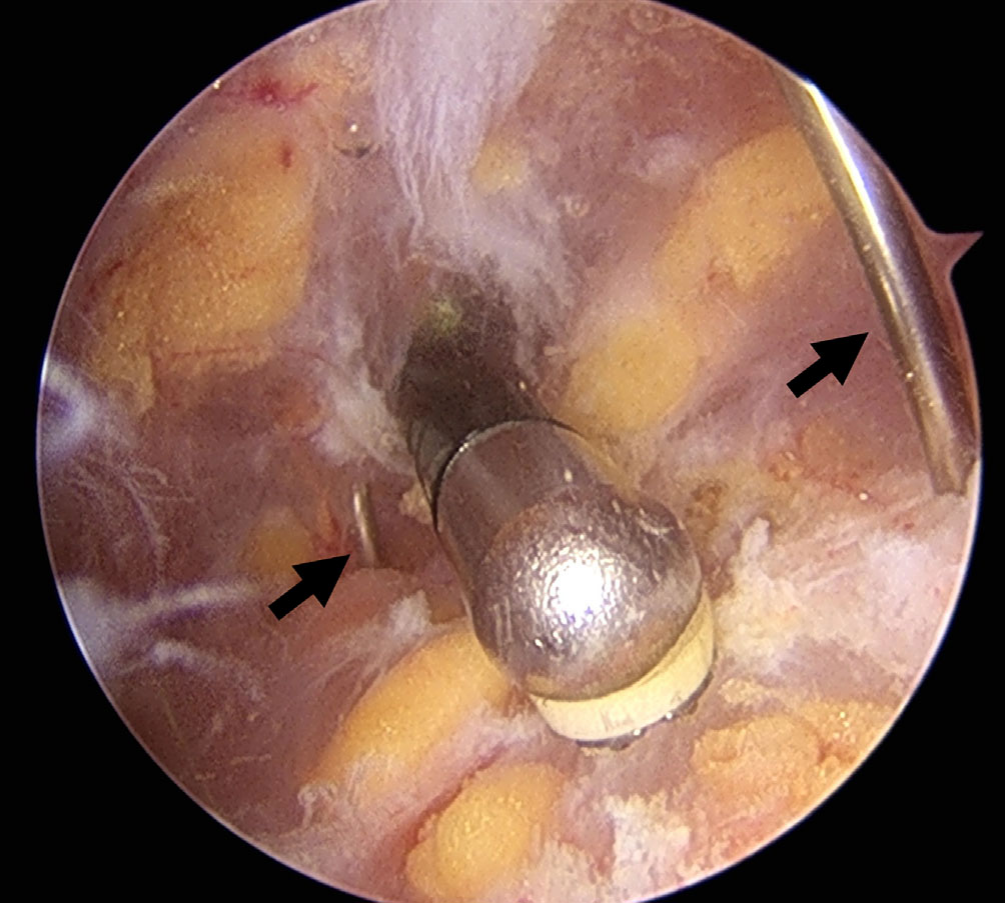
Arthroscopic view showing confirmation of the site using a spinal needle (arrows).

The surgery was performed by two experienced hip arthroscopic surgeons who are professors at the university hospitals. All patients underwent surgery in the lateral decubitus position, similar to the position used for hip replacement surgery. All surgeries were performed under general anesthesia. All procedures were performed according to the technique previously described by Ilizaliturri *et al*. and Yoon *et al*.^9^, ^12^ The GT was identified proximally and the vastus lateralis ridge distally as anatomical landmarks for portal placement (Figure 1). The proximal portal was placed three fingerbreadths above the tip of the GT, and the distal portal was placed 1 cm below the vastus lateralis ridge. To ensure that the area of snapping could be positioned between the two portals and to obtain adequate working length to access the snapping zone, we adjusted the portal entry points based on the patient's physique. Following a stab incision of the skin, a trocar was introduced through each portal between the subcutaneous fat layer and ITB. Gentle and meticulous blunt dissection was performed using a trocar, and the space was maintained using a water pump. Using a shaver and high‐frequency ablator, we exposed the snapping area. A standard 4‐mm 30° arthroscope was used. Flexion‐extension and adduction‐abduction movements of the hip were performed to arthroscopically confirm the margins of the snapping area. Using a spinal needle, we matched the margin to the previously created skin markings (Figure 2). Subsequently, ITB release was performed by initially creating a parallel incision using a high‐frequency ablator, following vertical extension, and finally creating a diamond‐shaped defect with meticulous hemostasis until snapping disappeared.

### Management of Residual Snapping after Extensive ITB Release

To exclude adjustable potential factors related to residual snapping, we followed the protocol based on a systematic order: (i) ITB release was performed, ensuring sufficient release within the arthroscope's field of view. Intraoperative hip range of motion were performed to confirm the absence of residual thickening. Attention was particularly paid to releasing the posterior edge, as residual thickening could persist in this area.^9^ (ii) In cases where a hyperemic trochanteric bursa was identified, trochanteric bursectomy was performed. (iii) In cases presenting with adduction difficulty and suspected tightness of the gluteus maximus, additional gluteal sling release procedure was performed with caution for potential vascular injury.^12^ (iv) If snapping persisted despite the absence of visually confirmed residual thickening after steps (i), (ii), and (iii), “persistence of snapping” was considered.

### Postoperative Management

Full weight‐bearing was permitted on postoperative day 1; however, the patients used crutches to support weight‐bearing and minimize pain during ambulation. During hospitalization, a continuous passive motion (CPM) device was employed to prevent soft tissue adhesion. The CPM protocol commenced the day after surgery without restricting the range of motion. The protocol was uniformly applied to all patients, involving two sessions per day, each lasting 30 min. The patients were instructed to ambulate using crutches for a month, and active abduction exercises were prohibited for 6 weeks postoperatively.

### Incomplete Release (Persistence of Snapping)

Persistence of snapping was observed in three patients even after sufficient resection of the snapping area, which was marked pre‐ and intraoperatively. Although definitive symptomatic improvement was observed in all three cases, intermittent palpable popping occurred with abduction‐adduction movements or full flexion‐extension exercises of the hip after deflation of the working space. In all cases, we extended the diamond‐shaped resection as far as possible within the visual field of the initial two portals instead of conversion to open surgery. The extension area did not extend over the area of either portal.

### Data Collection

We retrospectively collected demographic data, including sex, age, height, weight, BMI, and symptom duration. In addition, the data of the patients having recurrent symptomatic snapping converted to open surgery were collected as primary outcomes after a minimum 2‐year follow‐up. The visual analogue scale (VAS) and modified Harris hip (mHHS) scores were measured as secondary outcomes. All surgeries were performed by a single surgeon using the same surgical technique. Peri‐operative complications, including neurovascular injury and hematoma, were evaluated after using information from the patients' medical records. All continuous data are expressed as mean and standard deviation.

## Results

The mean age of the 33 (three female and 30 male) patients was 29.5 years (range: 19–61 years). Of the 34 hips, 18 were on the right side, and 16 were on the left side. One patient underwent surgery with bilateral hips. Table 1 summarizes the details of patients who defined the incomplete release. “Incomplete release” was identified in three of the 34 hips (8.9%). All three patients were male with an average age of 28.3 years (range: 20–41 years). All three patients who were diagnosed with incomplete release were followed up for at least 2 years. Cases of recurrent symptomatic snapping or conversion to open surgery were not observed. The symptoms of snapping hip syndrome spontaneously disappeared following routine postoperative rehabilitation in all cases. All patients presented improved VAS (4 ± 1) and mHHS (17 ± 6) scores. One patient presented surgical site hematoma as a postoperative complication.

**TABLE 1:** The patient demographics and clinical results.

Case	Sex	Age (years)	Height (cm)	Weight (kg)	BMI (kg/m^2^)	Symptom duration (months)	VAS preop	VAS final	mHHS preop	mHHS final	Duration of postop snapping disappearance	Recurrence	Perioperative Complications
1	M	24	187	98	28.0	10	5	0	69	91	1 mo f/u	none	none
2	M	41	180	80	24.7	24	3	0	70	89	3 mo f/u	none	none
3	M	20	182	72	21.7	12	5	1	74	85	1 mo f/u	none	hematoma

Abbreviations: BMI, body mass index; mHHS; modified Harris hip score; postop, postoperative; preop, preoperative; VAS, visual analogue scale.

### Case 1

A 24‐year‐old military recruit with a muscular build visited our outpatient clinic with a 10‐month history of snapping over his GT area with lateral pain, particularly when climbing a mountain or going up and down stairs. He was 187 cm tall and weighed 98 kg, and his body mass index (BMI) was 28.0 kg/m^2^. His pain continued despite conservative management, including medication, rest, and stretching exercises over 6 months. He wished to undergo surgical treatment, and we decided to perform arthroscopic ITB release.

Surgery was performed in the lateral decubitus position using two standard portals. Preoperatively, we accurately identified the snapping area over the GT and marked this zone using a surgical marker (Figure 1). ITB release was performed using a high‐frequency ablator and a shaver. Following the procedure, we used a spinal needle to appropriately match the skin marking with the arthroscopic resection margin. After confirmation of complete resection, abduction‐adduction and flexion‐extension exercises were performed intraoperatively to confirm the disappearance of snapping. Mild snapping persisted despite maximal resection extension using the initial portal. After reconfirming that the resection margin was adequately large compared with the initially marked area, we completed the surgery, although mild snapping persisted.

**FIGURE 3:**
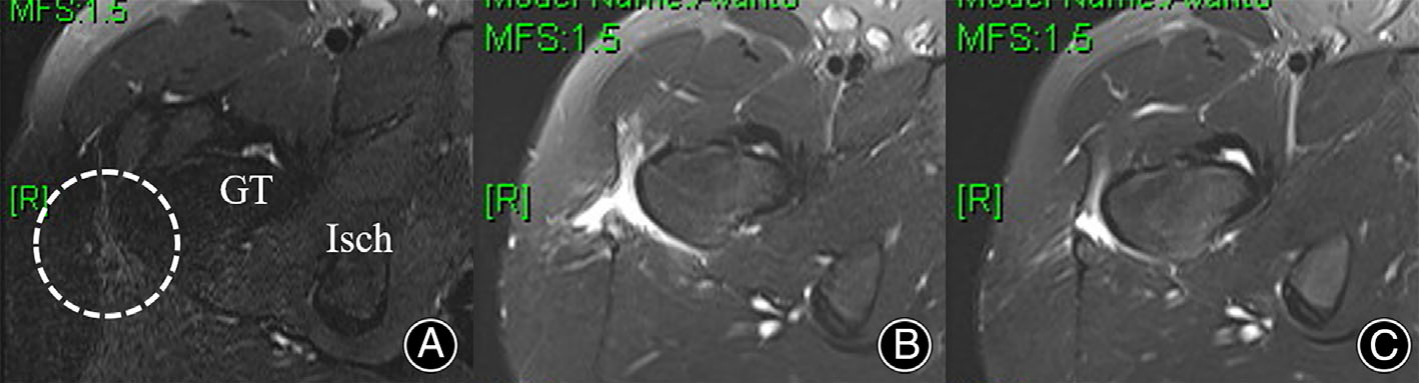
Preoperative axial T2‐weighted magnetic resonance image showing focal thickening and calcification of the anterior edge of the gluteus maximus (A, circle). Postoperative image showing that the lesion involving the GT of the femur has been adequately released (B). Postoperative 3‐month follow‐up image showing adequate healing of the resected area (C). GT, greater trochanter; Isch, ischium.

The usual postoperative protocol was as previously described. The patient could walk without a sensation of snapping on postoperative day 1. He was discharged on postoperative day 3 and underwent follow‐up at 1, 3, 6, 12 months and 2 years postoperatively. Physical examination performed at each visit, beginning with his 1‐month postoperative visit, did not reveal any snapping on palpation of the operated hip. The mHHS increased from 69 preoperatively to 91 at the 3 month postoperative follow‐up. His VAS score improved from 5 preoperatively to 0 at the 3 month postoperative follow‐up. Follow‐up magnetic resonance imaging showed a complete release of the snapping area. No pain or symptom recurrence was observed during the final 2‐year follow‐up (Figure 3).

### Case 2

A 41‐year‐old military sergeant presented with a 2‐year history of right hip snapping with intermittent pain. He underwent conservative management for over 1 year at another hospital. He did not report severe lateral hip pain upon presentation to our hospital; therefore, we initially decided to treat him conservatively. Owing to the persistence of symptoms at the 3 month follow‐up visit, he wished to undergo surgical treatment. He was 180 cm tall and weighed 80 kg, with a BMI of 24.7 kg/m^2^.

The surgical technique used was similar to that described previously, using two standard portals. Preoperatively, the snapping area was carefully demarcated using a surgical marker. Snapping persisted postoperatively despite complete resection of the snapping area, and extended resection was performed. We performed maximal resection of the snapping area with GT bursectomy secondary to thickening and hyperemia; however, mild snapping persisted intra‐operatively. The surgery was completed without conversion to open ITB release.

**FIGURE 4:**
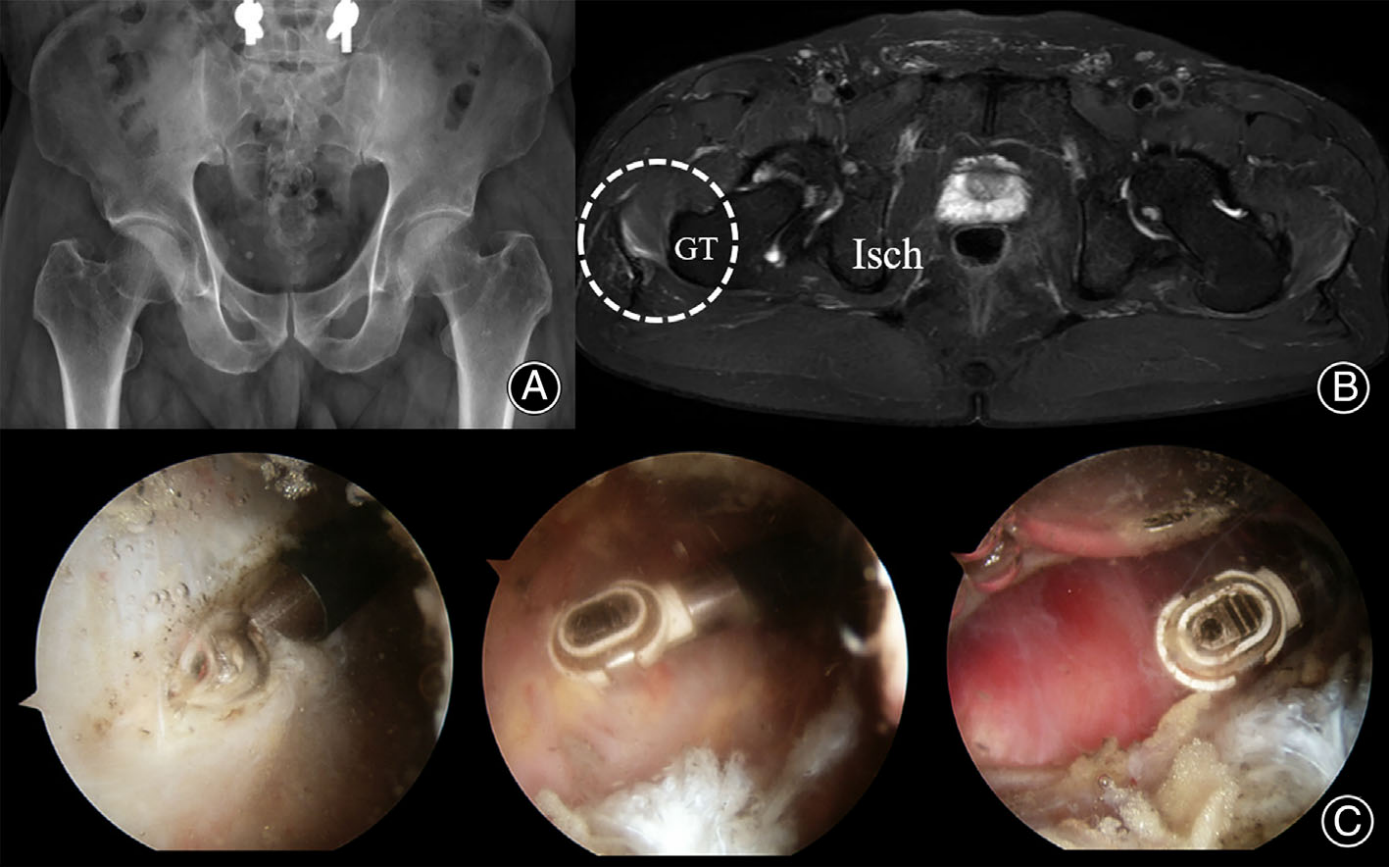
The pre‐ / post‐operative images and arthroscopic finding of 41‐year‐old male patient who underwent arthroscopic iliotibial band release. The simple hip X‐ray covers the symptomatic region (A), the T_2_‐weighted magnetic resonance image show the signal change between iliotibial band and GT on right hip (B, circle), and arthroscopic images show maximal arthroscopic resection with GT bursectomy (C), but the mild snapping persisted intra‐operatively. GT, greater trochanter.

During hospitalization, the patient did not complain of any residual snapping, and he was discharged without any postoperative complications. History‐taking and physical examination performed at the 3‐month postoperative follow‐up revealed no evidence of snapping. He was asymptomatic, even at the final 1‐year follow‐up. The mHHS increased from 70 preoperatively to 89 at the final follow‐up, and the VAS score improved from 3 preoperatively to 0 at the final follow‐up (Figure 4).

### Case 3

A 20‐year‐old military recruit presented to our outpatient clinic with a 1 year history of prolonged lateral hip pain and snapping. He underwent conservative management; however, participation in continuous military drills led to worsening pain and snapping. The mHHS and VAS score were 74 and 5, respectively. After a 3‐month trial of conservative treatment, the patient wished to undergo arthroscopic treatment, and surgery was performed.

The snapping area was marked preoperatively, and ITB release was performed after matching the skin markings and palpation during intraoperative hip manipulation. Popping did not disappear completely even after complete resection of the thickened ITB and the anterior portion of the gluteus maximus, which snapped upon examination. Additional release was performed with particular attention to the margin of the anterior gluteus maximus. Even with maximal resection (as much as possible corresponding to the working length of the ablator), snapping was not completely resolved. However, conversion to open surgery was not required.

**FIGURE 5:**
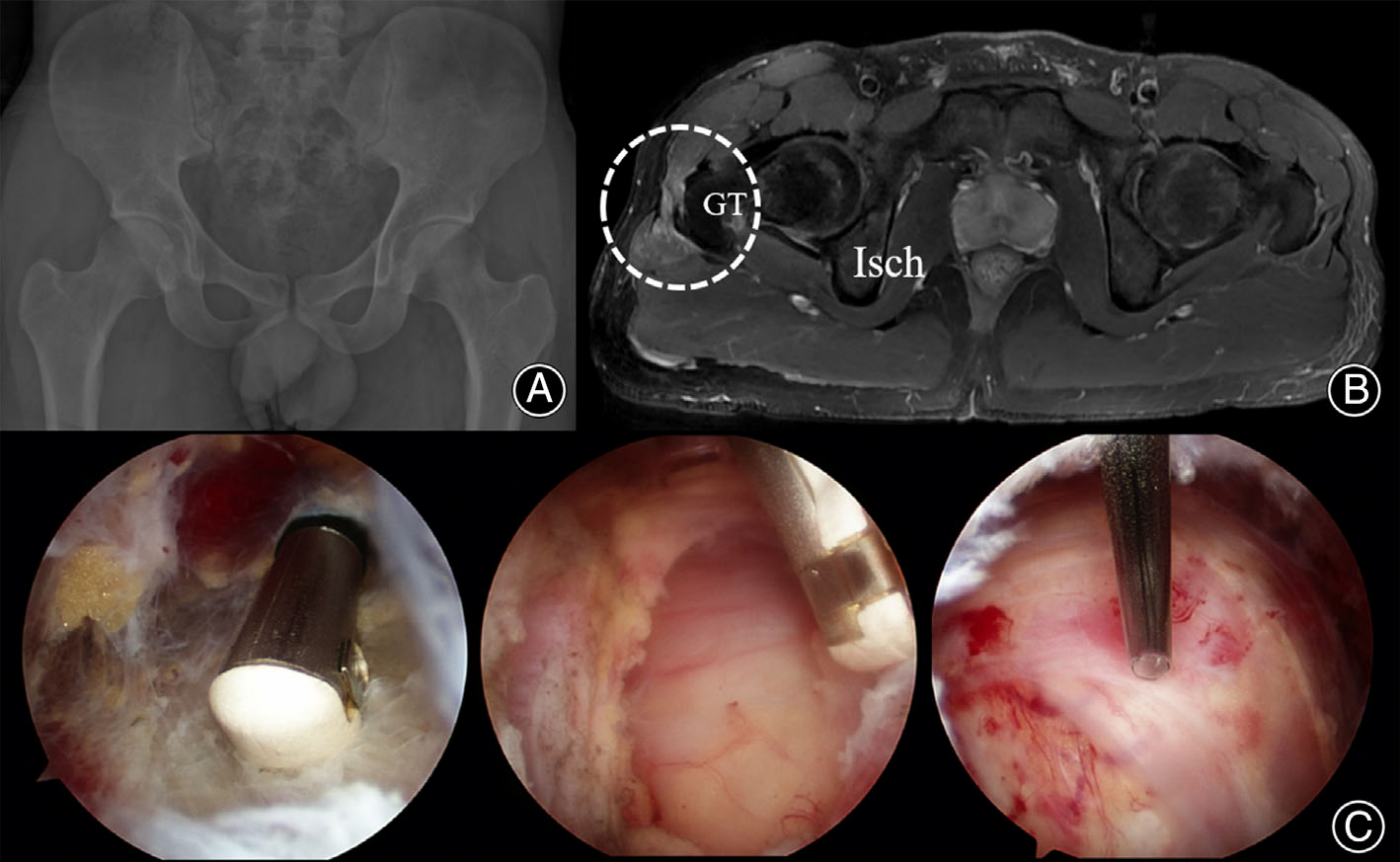
The pre‐/post‐operative images and arthroscopic finding of 20‐year‐old male patient who underwent arthroscopic iliotibial band release. The simple hip X‐ray covers the symptomatic region (A), the T_2_‐weighted magnetic resonance image show the signal change between iliotibial band and GT on right hip (B, circle), and arthroscopic images show maximal arthroscopic resection with GT bursectomy (C), but the snapping was not completely resolved. GT, greater trochanter.

On postoperative day 1, swelling was observed at the surgical site. A hypoechoic mass was identified on ultrasonography; postoperative hematoma was diagnosed, and a compressive dressing was applied. The hematoma was not aggravated during the 2 weeks of hospitalization; thus, the patient was discharged. Physical examination performed at the 1‐month postoperative follow‐up showed complete disappearance of snapping, and the patient reported no subjective snapping sensation. The hematoma completely subsided. No symptom recurrence was observed during the final follow‐up. The mHHS was 85, and the VAS score was 1 at the 2 year postoperative follow‐up (Figure 5).

## Discussion

The present study focused on the clinical outcome of patients diagnosed with ESH syndrome treated with arthroscopic “incomplete release.” As recurrent symptomatic napping was not observed, open surgery was not needed. In addition, the VAS and mHHS scores improved in all the three patients. Although refractory ESH syndrome was identified during arthroscopic surgery, residual symptoms are expected to be resolved without the need for open surgery.

### Clinical Outcome of Present Study Compared with Historical Data

Recently, Chu *et al*. reported promising long‐term clinical outcome for patients with ESH. There was increased functional score, and neither recurrence of snaps nor complications were recorded. However, there was no mention for incomplete arthroscopic release.^15^ Zhang *et al*. reported outstanding results of their three‐step arthroscopic release procedure for failed primary open gluteal muscle contracture release.^8^ They evaluated 136 cases of arthroscopic release as a solution for failed open gluteal muscle contracture release for revision surgery. However, no previous studies have described the natural course of incomplete release of ESH syndrome, especially following arthroscopic surgery. In the present study, persistent snapping was observed in >10% of all the arthroscopic ITB release cases during the study period. However, we showed a positive natural course of incomplete release of primary arthroscopic release surgery, not revision surgery, without any additional procedures related to gluteus medius and minimus, external rotator, or capsular release. In our opinion, this difference might be caused by the characteristics of the enrolled patients. The patients investigated in the study by Zhang *et al*.^8^ had gluteal muscle contracture due to failed open surgery, while our cases were not revision cases but primary surgery cases that did not respond to conservative management. Therefore, we believe that our study findings can be helpful in the decision‐making among surgeons with concerns on residual snapping symptoms following arthroscopic ITB release as a primary option.

### Difference between Arthroscopic Release of ESH Syndrome and Intra‐articular Hip Procedures

Notably, arthroscopic surgery for ESH syndrome differs from other intra‐articular hip arthroscopic procedures in that the former is performed in a soft tissue plane present over or beneath the ITB‐gluteus maximus complex. Thus, the operating time is longer, and maneuvering arthroscopic instruments is more difficult secondary to excessive expansion of the working space. This difficulty is more pronounced in obese patients or in those with a large body build.

### Concerns for Conversion to Open Surgery

In patients who show persistence of snapping following arthroscopic intervention, surgeons might consider conversion to open surgery. However, conversion to open surgery is not an easy decision because the latter is associated with cosmetic issues. Additionally, convincing the patient postoperatively regarding the justification for conversion is difficult.

### Importance of Appropriate ITB Release and Systematic Assessment of Hip Motion

In this case series, we observed complete symptom resolution without snapping recurrence during the minimum 2 year follow‐up in all three cases. In our opinion, we can expect symptom resolution without the need to convert to open surgery once we perform appropriate ITB release and remove the primary cause of snapping appropriately. We believe that if the hip motion is sufficiently assessed in a systematic order, ensuring thorough resolution of thickened areas, especially posterior thickening, and if bursectomy is adequately performed, even in cases where residual snapping persists after a regular ITB release, one can anticipate natural symptom relief. Therefore, when surgeons perform arthroscopic ITB release appropriately, patients may expect spontaneous resolution in the postoperative course, even if residual snapping persists. Hence, it is advisable to favor arthroscopic surgery over open surgery whenever possible.

### Gluteal Sling Release

“Gluteal sling release” is an alternative arthroscopic procedure used to treat ESH syndrome. This technique involves the release of the femoral insertion of the gluteus maximus tendon and was introduced by some authors as an additional technique to release hip adduction contractures.^12^ It is also useful as an alternative technique in patients with defects of the ITB.^16^ In this study, we selected the conventional arthroscopic ITB release technique for all cases. Subsequently, two patients exhibited persistent tightness of the gluteus maximus. An additional gluteal sling release procedure was performed for these two patients, and gluteal sling ossification was observed in one of them. However, none of the three patients who continued to experience persistent snapping exhibited adduction contracture or tightness of the gluteus maximus. Therefore, gluteal sling release was not considered an additional procedure for the three patients classified as having “incomplete release” with persistent symptoms intraoperatively.

### Limitations and Prospect

The limitations of this study are as follows: first, the sample size was relatively small. Following an increase in the population engaging in sports, the risk of ESH syndrome has also increased. However, considering that conservative treatment is the first‐line treatment for ESH syndrome and that a relatively small number of patients need surgical treatment, this report is meaningful even though a small number of cases were evaluated. Large‐scale studies are necessary in the future to gain a better understanding of this subject. Second, several arthroscopic techniques have been introduced for ESH syndrome; however, in this case series, we analyzed a single technique for ITB release. Further studies are required to investigate other techniques in this context.

## Conclusions

When refractory ESH syndrome is identified during arthroscopic surgery, appropriate ITB release and removal of the major lesion causing snapping are expected to lead to resolution of residual symptoms without conversion to open surgery.

## Conflict of Interest Statement

The authors declare no conflict of interest.

## Ethical Statement

The study was conducted in accordance with the Declaration of Helsinki, and approved by the Institutional Review Board of Asan Medical Center (IRB No. 2024‐0050).

## Author Contributions

Investigation, data curation, writing—review and editing E.J.L.; validation, supervision J.W.K.; data curation, editing C.H.D.; writing—original draft preparation, conceptualization, methodology C.‐H.K. All authors have read and agreed to the published version of the manuscript.

## Funding Information

This work was supported by the Korea Evaluation Institute of Industrial Technology grant funded by the Ministry of Trade, Industry and Energy (MOTIE, Korea) (No. 20016543, Development of titanium alloy wire rod with roundness of 50 μm or less for biomedical and dental/orthopedic implant application technology).

## Authorship Declaration

All authors have read and agreed to the published version of the manuscript.
